# Navigating Gravity: Competing Effects Result in Opposing Taxis for Different Janus Swimmers

**DOI:** 10.1002/smll.202508984

**Published:** 2025-12-28

**Authors:** Amir Sheikh Shoaei, Jens‐Uwe Sommer, Juliane Simmchen

**Affiliations:** ^1^ Department of Physical Chemistry Technische Universität Dresden Dresden Germany; ^2^ Department of Physics Technische Universität Dresden Dresden Germany; ^3^ Department of Pure and Applied Chemistry University of Strathclyde Glasgow UK

**Keywords:** gravitaxis, micro‐galton board, micromotors, particle sorting

## Abstract

Microorganisms often employ tactic behaviors as a survival strategy, enabling them to move in response to different stimuli. One fascinating example is the use of gravity for their orientation, where gravity provides a directional cue for swimming. Separately, when microswimmers move near solid surfaces, hydrodynamic interactions with the wall can alter their trajectories and reorientation dynamics.

This study investigates the combined effects of gravitational torque and wall‐induced hydrodynamics on the orientation and movement of Janus colloids coated with either platinum or copper, swimming toward their inert side (Pt) or the catalytic half (Cu), respectively. Both particle types exhibit alignment with the gravitational direction on inclined substrates due to bottom‐heaviness; however, Cu@SiO_2_ particles, with lighter metal caps, show a significantly narrower orientation distribution compared to Pt@SiO_2_ particles. This enhanced alignment is attributed to the distinct hydrodynamic coupling of catalytic‐forward type swimmers near boundaries.

Using these differences, a simple yet effective method for separating active particles based on their gravitactic behavior is presented. Furthermore, we demonstrate that active and passive particles can develop a lateral distribution analogous to that of a Galton board, highlighting potential applications in educational tools and microfluidic‐based computational systems.

## Graphical abstract

The combined effects of gravity and wall‐induced hydrodynamics on the motion of Janus colloids are investigated. Platinum‐ and copper‐coated silica microswimmers show distinct gravitactic alignment near surfaces due to different hydrodynamic couplings. This distinct gravitactic behavior enables separation of initially mixed active particles.

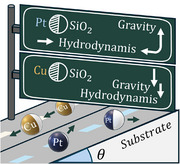

## 1 Introduction

For humans, gravity is so pervasive in our lives and so deeply ingrained in our perception of the world that we often fail to note its fundamental influence [1, 2]. Many motile organisms, however, use it to orient and swim preferentially in the direction defined by gravity, i.e., exhibiting gravitaxis [3]. For instance, the upward vertical migration of Phytoplankton species, i.e., negative gravitaxis, during the day gives them access to daylight while they do positive gravitaxis at night to obtain nutrients [4, 5]. Different organisms, such as motile *C. elegans* nematodes show positive gravitaxis by aligning their swimming motion with gravity direction [3, 6]. These responses are mechanistically diverse and not always fully understood [3, 7]. Moreover, often micro‐organisms are exposed to more than one stimulus. Kessler [8] showed that algae cells swimming in Poiseuille flow, which is aligned with the direction of gravity, accumulate either in the center or around the walls of the pipe, depending on whether the direction of the flow is opposite or the same as gravity. He explained that the balance between gravitational torque and hydrodynamic torque, due to spatial variation of the velocity, results in a stable orientation of the swimmer.

Recent research shows that active colloids can mimic the behavior of their biological counterparts [9, 10]. Among active colloids, Janus structures are widely utilized due to their ability to generate asymmetric physicochemical environments, such as chemical gradients, that enable autonomous motion. A prototypical example of a Janus particle is a passive core half‐coated with platinum, which catalyzes hydrogen peroxide to generate chemical gradients that drive motion [11]. Thereby, the speed of particles increases with the increase in the $H_{2}O_{2}$ concentration [11, 12]. For platinum‐coated particles, the particle image velocimetry (PIV) analysis indicates a pusher‐type propulsion mechanism in line with their cap‐backward motion [13]. The Pt Janus particles can also navigate guided by external stimuli: For instance, in a shear flow, Katuri et al. [14] showed that Pt Janus particles reorient normal to the flow and move across it. Additionally, optical tweezers can be used to impose controlled external torques and forces on colloidal particles, enabling direct quantification of the force balances that govern their motion [15, 16]. Recently, Raj et al. employed optical tweezers to measure the self‐diffusiophoretic propulsion force generated by Janus particles with different platinum patch coverages [17]. Their measurements revealed that particles with smaller catalytic patches generate larger propulsion forces in bulk fluid, consistent with the observation that they also swim faster.

Of special interest is gravitactic behavior of microswimmers. Janus particles typically possess a catalytic metallic cap, resulting in an inhomogeneous mass distribution that induces a preferential orientation along the direction of gravity [9, 10]. In an early work, Campbell and Ebbens [18] reported that platinum‐coated polystyrene colloids move upward, away from the bottom substrate and against gravity. Ginot et al. [19] used gold Janus particles half‐coated with platinum to study the sedimentation density profile where the sedimentation speed is comparable to the propulsion speed. Wolff et al. [20] derived analytical expressions for sedimentation length and mean particle orientation of active colloids with and without bottom heaviness. They found that bottom heaviness increases the orientational order, whereas maximal orientational order occurs in intermediate swimming speeds for both cases. While negative gravitaxis is mostly studied for spherical active colloids, it is shown that bimetallic rods [21] and fore‐rear asymmetric active colloids [22] can also achieve that. The latter is studied using L‐shaped particles with homogeneous mass density.

In our recent work, we introduced half‐coated copper Janus particles that exhibit propulsion toward their catalytic cap. This is in contrast to platinum‐coated Janus particles, which move away from their cap [23]. Furthermore, Cu Janus particles exhibit distinctive behavior in shear flow, demonstrating negative rheotaxis by migrating against the flow direction, even at low shear rates. While PIV analysis with 1 μm sized tracers suggests a pusher nature for Pt@SiO_2_ particles [13], a methodically clean approach using smaller tracers has not been demonstrated yet, neither for Pt nor for Cu Janus particles. Nevertheless, we hypothesize that the flow fields generated by Cu and Pt Janus particles are fundamentally different, as suggested by multiple observations, including their phenomenologically different interactions with passive colloids [24] and distinct pairwise interaction dynamics [25].

Given the distinct behaviors of Pt@SiO_2_ and Cu@SiO_2_ particles under various environmental conditions, we aim to investigate the motion of both particle types under the influence of gravity on a tilted substrate, a configuration more commonly encountered in the real world than perfectly planar, horizontal surfaces. Theoretical [26, 27] and experimental [28] studies have shown that passive particles on inclined substrates exhibit coupled rotational and translational motion, as well as non‐trivial collective behavior [29]. Although some studies have employed inclined substrates to explore the effects of gravity on active particles [19, 21, 22], sedimentation effects are typically neglected.

In the present manuscript, we first confirm earlier observations of gravitactic behavior for Pt@SiO_2_ particles and then compare their behavior to Cu@SiO_2_ Janus particles. We show that although bottom‐heaviness reorients both particles in the direction defined by gravity, opposite tactic responses emerge from the distinct activity patterns. Additionally, Cu Janus particles exhibit stronger alignment compared to their Pt equivalents, despite the lower density of copper. After rationalising this behavior, we look at the application side and exploit the different gravitactic behaviors of these systems to separate a mixed suspension of active and passive particles of identical size. Furthermore, as the particles accumulate at the top or bottom of the container walls (depending on the positive or negative nature of their gravitaxis). We show that separating particles horizontally in different bins creates a setup resembling a micronscale Galton board, i.e., a device that resembles invented by Sir Francis Galton that visually demonstrates how a normal distribution originated from a random process.

## 2 Results and Discussion

### 2.1 Active Particles on Horizontal Substrates

**Figure 1:**
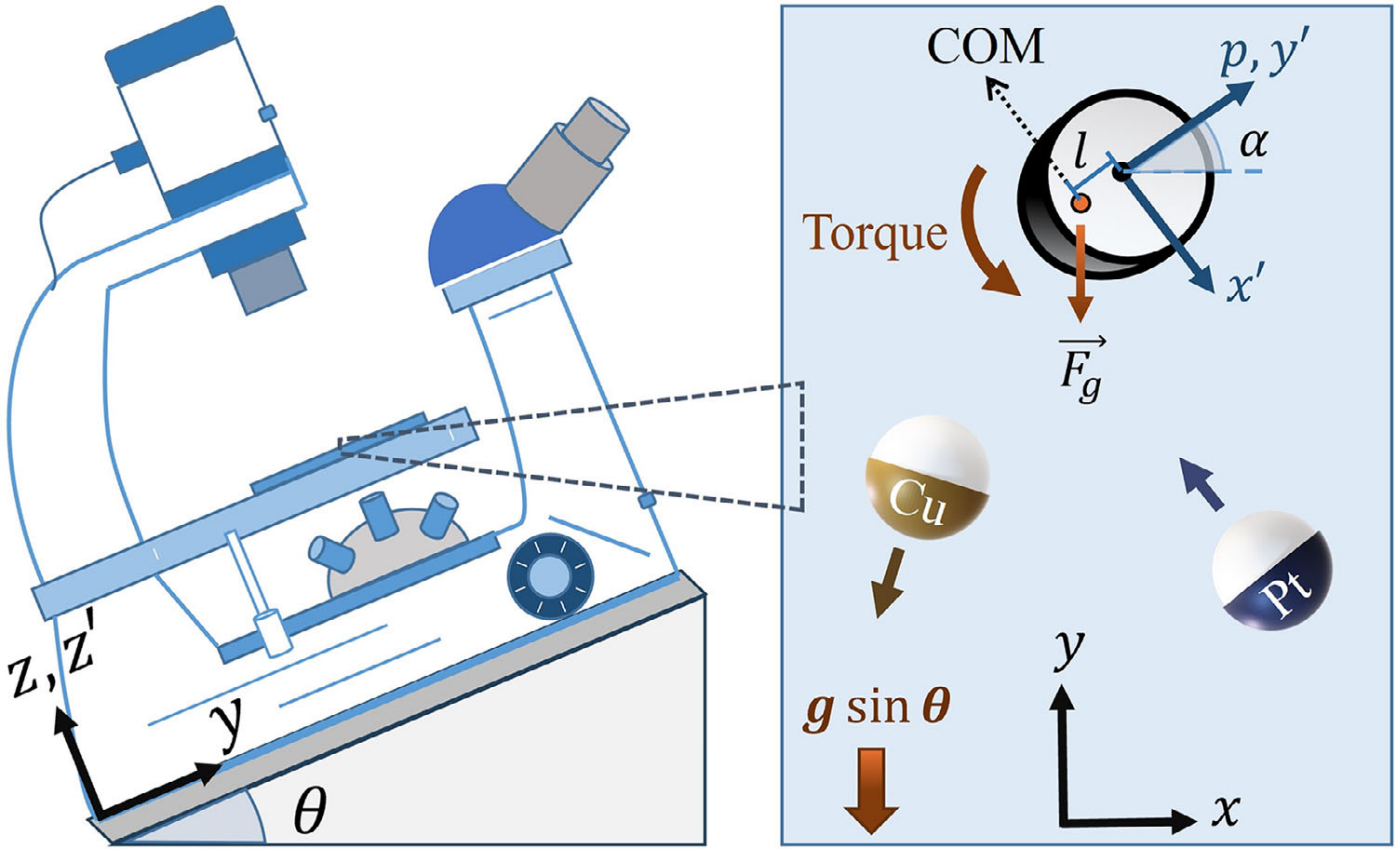
Schematic representation of the experimental setup. The inset shows a cross‐section of an active particle on the inclined substrate, along with its center of mass (COM), which is located at a distance ł from the geometric center.

**Figure 2:**
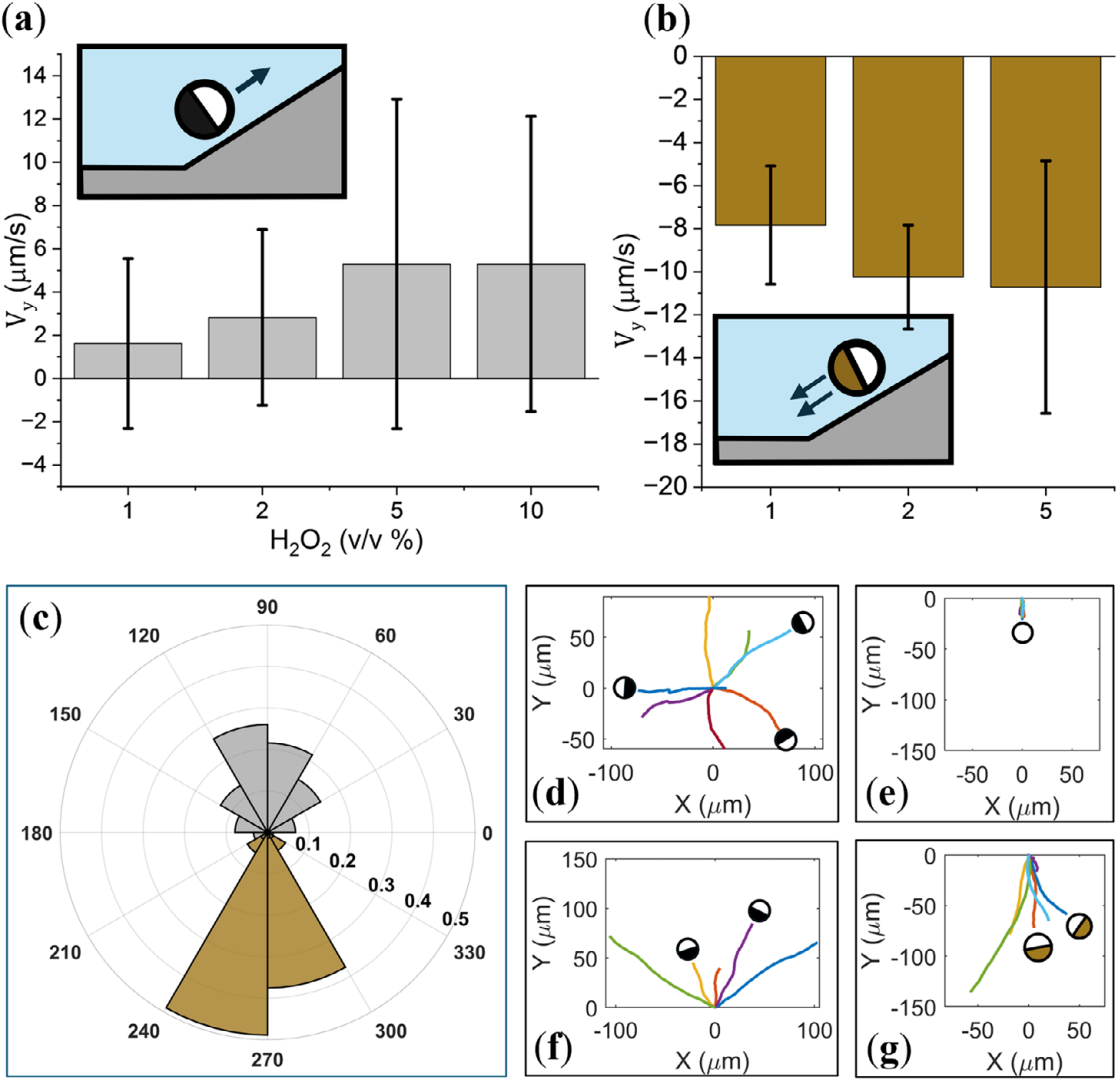
Average speed of (a) Pt@SiO_2_ and (b) Cu@SiO_2_ Janus particles in varying $H_{2}O_{2}$ concentrations. (C) Comparison between the in‐plane angle (α) distribution of Pt@SiO_2_ and Cu@SiO_2_.(d) Typical trajectory of active particles at horizontal substrates and (d) sedimentation trajectory of SiO_2_ particles on tilted substrate. Typical trajectories of (f) Pt@SiO_2_ and (g) Cu@SiO_2_ particles on a tilted substrate. Error bars represent the standard deviation.

We begin with the motion of the active particles close to a horizontal substrate. In our experiments, we use Janus particles composed of 5 μm silica cores, half coated with a layer of 30 nm platinum or copper, which acts as a catalyst for the degradation of a hydrogen peroxide fuel solution, leading to an inhomogeneous concentration of the reaction products around the particle. This anisotropy results in a surface flow around the particle, which leads to directed motion of the particle (Figure 2d). This motion increases with fuel availability, hence, with higher concentrations of peroxide, the swimmer moves faster (as shown for both types of particles in Supporting Information, Section S1). Besides the general perspective, two different propulsion mechanisms have been considered in previous studies for the two systems of Pt and Cu coated Janus particles [11, 23, 30]. For Pt Janus particles, the self‐established concentration gradient leads to self‐diffusiophoresis with an undefined proportion of ionic contributions [31]. On the other hand, a strong ionic contribution is assumed for the Cu Janus particles in our previous study [23]. In this model, the polar and near‐equatorial regions of the particle act as anode and cathode, respectively, due to the variations in the thickness of the copper layer on a silica core and the partial oxidation of copper by $H_{2}O_{2}$. While we have not measured the specific flow fields, we follow the nomenclature by Liebchen and Löwen and refer to the ‘catalytic forward’ moving swimmer as puller, as opposed to the inert forward swimmer as pusher. In both systems, the micromotor's direction p (the vector from catalytic toward inert pole shown in Figure 1) remains almost parallel to the substrate while moving, and Brownian motion mostly affects the direction in the x‐y plane.

### 2.2 Active Particles on a Tilted Substrate

To investigate the gravitactic behavior of the Cu and Pt‐coated Janus particles, we use a setup inspired by the Bechinger group [22]. We place the microscope on a triangular ramp with a defined angle $\theta =15^{\circ }$ that makes it possible to control the contribution of gravitational force on particles (Figure 1). Furthermore, compared to tilting the sample, this ensures that all particles are in the same focal plane, resulting in better image quality. Here we define our laboratory frame such that it coincides with the substrate and its y‐axis is aligned with the projection of the gravity vector on the wall's plane, as shown in Figure 1. In the absence of activity, particles slide down in the y direction (Figure 2e). The velocities of different particles sliding down, in the absence of self‐propulsion, are provided in Supporting Information Section S2.

Upon adding fuel, micromotors reorient parallel to the substrate, similar to moving on a horizontal plane. To characterize the particles' orientation, we define the in‐plane angle of the micromotor α representing the angle between particle direction and y axis (Figure 1). In both platinum and copper Janus particles, motion occurs preferentially parallel to the y direction, i.e., the direction defined by gravity (Figure 2f,g). This phenomenon, known as gravitaxis, is also reported in other studies for platinum Janus particles [18, 32, 33]. To see the bias induced by gravity in the motion of the micromotors, we measure the average velocity of the particles in different concentrations of the peroxide solution (Figure 2a,b). On average, the Pt Janus particles move against gravity, showing negative gravitaxis. Their velocity increases with the concentration of fuel (Figure 2a). In contrast, Cu Janus particles move in the negative y direction and show positive gravitaxis (Figure 2b). Their velocity also increases with fuel concentration, similar to Pt‐coated particles. However, because the fuel degradation on Cu is not a perfectly catalytic process and a low amount of ions are created, particles stick frequently at higher concentrations. Therefore, no value is shown for 10 % $H_{2}O_{2}$. These two distinct behaviors can be understood within the framework of the motion of bottom‐heavy active particles.

The inhomogeneous mass distribution of the Janus particles introduces a deviation length l between the center of mass and the hydrodynamic resistance of the Janus particle (Figure 1). This length can be calculated assuming an eggshell model for the coated part of the Janus particle as $l=0.75\;(r+0.5\;l_{thick})$, where r is the particle's radius, and $l_{thick}$ is the thickness of the cap (For further discussion regarding the model dependence of the possible values of l, please see Supporting Information, Section S10) [18]. As a result, gravity exerts a torque $T=m_{a}gl\sin\alpha \sin\theta$, which reorients particles in the direction defined by gravity, where $m_{a}$ and g are the mass of the active cap and gravitational acceleration, respectively. Subsequently, both Cu and Pt Janus particles move while reorienting with gravity, but they move in opposite directions: copper particles move with their catalytic cap forward, while the Pt Janus particles move with the cap facing backward (see Figure S3 in the Supporting Information). Hence, while their direction of movement relative to gravity depends on their intrinsic movement direction (toward the cap or away from the cap), the alignment with gravity should depend on the magnitude of the gravitational torque that reorients them.

The time evolution of the angle α is described by the Langevin equation

(1) $$\frac{d\alpha }{dt}=M_{R}T+\sqrt{2D_{R}}W\left(t\right),$$

where $M_{R}$ is the rotational mobility, T is the applied torque, and W(t) is Gaussian white noise with zero mean and correlation function $\left\langle W\left(t\right)W\left(t^{\prime }\right)\right\rangle =\delta \left(t-t^{\prime }\right)$. Since a certain amount of energy is required to rotate a bottom‐heavy particle to a particular orientation, we can use Boltzmann statistics to determine the stationary probability distribution function for the orientation of the Janus particles, which is given by [18, 21]:

(2) $$p\left(\alpha \right)=\frac{e^{k\sin\alpha }}{2\pi I_{0}\left(k\right)},$$

where $I_{0}$ is the modified Bessel function of order zero, and $k=T/k_{B}T$ shows the relative importance of gravitational torque compared to thermal fluctuations. Equation 2 shows a von Mises distribution, which is the circular analog of the normal distribution. The concentration parameter k, analogous to inverse variance, dictates how tightly the distribution is clustered around the mean direction, with larger values corresponding to higher peaks and k=0 resulting in a uniform distribution. For the Janus particles of the same size in an identical experimental condition, k linearly scales with the mass of their cap. The typical angle distribution of the movement orientation of the Pt and Cu micromotors is given in Figure 2c. It is apparent that the particles' orientation is, on average, aligned with the y axis for both systems. Surprisingly, Cu Janus particles show a stronger alignment with the direction of gravity, despite noticeably lower density of the copper ($8.96\;g/{\mathrm{cm}}^{3}$) compared to platinum ($21.45\;g/{\mathrm{cm}}^{3}$).

**Figure 3:**
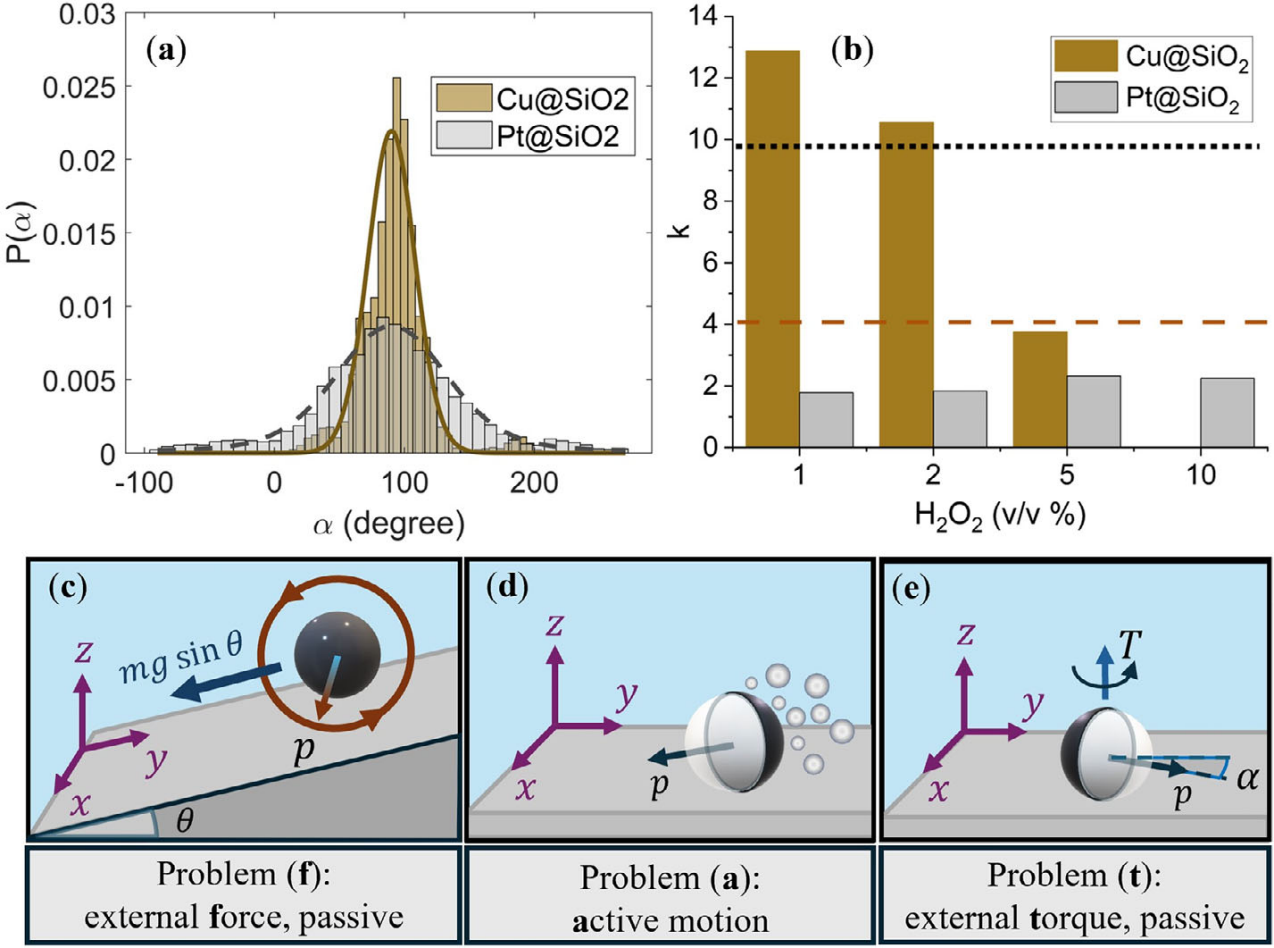
(a) In‐plane orientation angle (α) distribution of Pt@SiO_2_ and Cu@SiO_2_ Janus particles, along with their corresponding von Mises fitted curves, shown by the black dashed line and solid line, respectively. (c) Passive sphere sliding and rotating on a tilted substrate in an external force (problem (f)). Side view (d) and top view (e) of a bottom‐heavy, neutrally buoyant active particle on a tilted substrate (problem (a,t).

To quantitatively compare the orientational alignment behavior between the two systems of Pt and Cu particles, we find the von Mises distribution parameter k corresponding to the experimental data. Across all $H_{2}O_{2}$ concentrations, we observe a close agreement between the experimental distributions and their corresponding fitted curves. A typical distribution of the orientation and the corresponding fitting for Pt and Cu particles is shown in Figure 3a. Furthermore, Figure 3b shows the fitted concentration parameter k for Pt and Cu systems in different concentrations of $H_{2}O_{2}$. For all the fuel concentrations, Pt Janus particles show consistently lower values of k compared to their Cu coated counterparts, and their k value is almost 5 times less than their theoretically expected value (black dotted line). Conversely, the orientational alignment of Cu Janus particles is stronger than the theoretical expectation (brown dashed line), except in 5%(v/v), which has almost the same value.

As the orientational alignment occurs in response to gravitational cues, the tilt angle of the glass slide, θ, defines the magnitude of the aligning gravitational torque. Therefore, a decrease in θ is generally expected to reduce the concentration parameter k, and consequently to lower the average particle velocity in the y‐direction. This trend is observed for both types of Janus particles at the same $H_{2}O_{2}$ concentration, as shown in Supporting Information Section S8.

#### 2.2.1 Dynamical Model

To explain the discrepancy between the theoretical model for bottom‐heavy micromotors and our experimental data, we consider the contribution from hydrodynamic interactions of the particle and the wall. First, we note that our system is in the low Reynolds number regime ($Re\;\approx 5\times 10^{-5}$), and the Stokes equations can be applied to describe the hydrodynamics of the system. Due to the linearity of the Stokes' equations, we can consider the contributions of external *force* (f), external *torque* (t), and the particle's *activity* (a) independently and superimpose them. Therefore, according to each of the contributions, the flow field around the bottom‐heavy active particle can be expressed as a combination of three simpler problems, namely, a passive heavy particle on a tilted substrate (problem (f)), a buoyant active particle close to a wall (problem (a)), and a buoyant bottom‐heavy passive particle on a tilted substrate (problem (t)). A schematic representation of the problems (f), (a), and (p) is shown in Figure 3c–e. Subsequently, by superimposing the translational and angular velocities of each problem, we can obtain the dynamics of the original systems as $U=U^{\left(f\right)}+U^{\left(a\right)}+U^{\left(t\right)}$ and $\Omega =\Omega^{\left(f\right)}+\Omega^{\left(a\right)}+\Omega^{\left(t\right)}$. Here, U and Ω are translational and angular velocities, respectively, and the superscripts refer to corresponding problems. The velocity of the orientation vector is obtained by $\dot{p}=\Omega \times p$. In the following, we will demonstrate that even in the absence of aligning torque, we expect the active particles to assume a fixed orientation in the presence of an external force (combination of the problems (f) and (a), which we refer to as problem (f,a)). Because of the similarities between the problem (f,a) and the problem of active particles in shear flow, we use a similar approach as references [14], and [34] to construct our dynamical model. We first proceed to analyse the dynamics arising from problem (f).

If the bottom substrate is horizontal, the gravitational force on the particle is counteracted by electrostatic repulsion from the wall, suspending the particle near the substrate. Close to the tilted substrate, gravity has an extra contribution parallel to the substrate, which moves the particle parallel to the wall. This constitutes a classical hydrodynamic problem [26, 27, 35, 36], which has been followed more recently by adding more complexity to the model, such as including a Navier slip condition [37] for the wall. The applied force spins the particle in the $\hat{x}$ direction, normal to the wall direction and the direction of the applied force, as the frictional force is higher on the side of the particle close to the wall. Following the asymptotic solutions of Goldman et al. [26], the translational and angular velocity of the particle will take the form:

(3) $$\begin{array}{cc} \Omega^{\left(f\right)} & =-\frac{U^{\left(f\right)}}{r}f\left(h/r\right)\hat{x}, \end{array}$$

(4) $$\begin{array}{cc} U^{\left(f\right)} & =\frac{F^{\left(f\right)}}{6\pi \eta r}\;g\left(h/r\right)\hat{y}, \end{array}$$

where η is the dynamic viscosity of the fluid, h is the height of the particles' center from the substrate, $F^{\left(f\right)}=-\left(m_{particle}-m_{fluid}\right)g\sin\theta$ is the gravitational force acting on the particle ($m_{particle}$ and $m_{fluid}$ are the mass of the particle and mass of the water with the same volume as the particle, respectively) and the functions f(h/r) and g(h/r) represent the influence of hydrodynamic interaction between the colloid and the wall and are provided in Supporting Information Section S4. The particle's rotational velocity is proportional to its sedimentation velocity (on the tilted wall), which can be measured experimentally. Therefore, the rotational velocity can be adjusted by changing the effective gravitational force on the particle (for example, by changing the slope of the wall or the density of the particle). f(h/r) is a monotonic continuous function, where f(h/r)→0 as h/r→∞ (corresponding to the particle in the bulk), and f(h/r)→0.25 as h/r→1. Moreover, g(h/r)→1 as h/r→∞, and $g\left(h/r\right)∼\frac{2}{\ln(h-r)/r}$ as h/r→1. Since the particle rotates as it moves, the tip of the orientation vector p follows a circle in z‐y plane

(5) $${\dot{p}}^{\left(f\right)}=\Omega^{\left(f\right)}\times p^{\left(f\right)}=\frac{U^{\left(f\right)}}{r}f\left(h/r\right)\left(p_{y}\hat{z}-p_{z}\hat{y}\right)$$

Therefore, the component $p_{x}$ remains constant and the speed of the tip is proportional to the radius of the circular path $\sqrt{1-p_{x}^{2}}$.

We now analyze the motion of an active particle moving near a horizontal substrate. For the sake of simplicity, we describe the dynamics of the particle in the coordinate system $x^{\prime }y^{\prime }z^{\prime }$, shown in Figure 1, where $z^{\prime }$ has the same direction as z and $y^{\prime }$ lies in the symmetry plane of the particle defined by $\hat{z}$ and p. Note that the unit vectors $\hat{x^{\prime }}$ and $\hat{y^{\prime }}$ can be derived as $\hat{x^{\prime }}=\left(p_{y}\hat{x}-p_{x}\hat{y}\right)/\sqrt{1-p_{z}^{2}}$, and $\hat{y^{\prime }}=\left(p_{x}\hat{x}+p_{y}\hat{y}\right)/\sqrt{1-p_{z}^{2}}$. Assuming that the particle has axisymmetric geometry and activity, the particle can only move in the plane of the symmetry ($U_{{\hat{x}}^{\prime }}^{\left(a\right)}=0$), and the rotation of the particle is restricted in the ${\hat{x}}^{\prime }$ direction ($\Omega_{{\hat{y}}^{\prime }}^{\left(a\right)}=\Omega_{{\hat{z}}^{\prime }}^{\left(a\right)}=0$). Therefore, the rotational velocity of the particle can be described by the function $\Omega_{{\hat{x}}^{\prime }}^{\left(a\right)}\left(p_{z^{\prime }},\frac{h}{a}\right)$. where the details of the function depend on the intrinsic properties of the particle and its interaction with the wall (note that $p_{z}=p_{z^{\prime }}$). Now, we can combine the contributions from problems (f) and (b) to describe the dynamics of the motion of an active particle in the presence of an external force (problem (f,a)). The equations for the evolution of the direction of the particle ${\dot{p}}^{\left(f,a\right)}=\Omega^{\left(f,a\right)}\times p^{\left(f,a\right)}$ and the height of the particle $\dot{h}=U_{z}^{\left(f,a\right)}$ read

(6) $$\begin{array}{cc} {\dot{p}}_{x}^{\left(f,a\right)} & =-\frac{\Omega_{{\hat{x}}^{\prime }}^{\left(a\right)}p_{x}p_{z}}{\sqrt{1-p_{z}^{2}}}, \end{array}$$

(7) $$\begin{array}{cc} {\dot{p}}_{y}^{\left(f,a\right)} & =\frac{U^{\left(p\right)}}{r}f\left(h/r\right)p_{z}-\frac{\Omega_{{\hat{x}}^{\prime }}^{\left(a\right)}p_{y}p_{z}}{\sqrt{1-p_{z}^{2}}}, \end{array}$$

(8) $$\begin{array}{cc} {\dot{p}}_{z}^{\left(f,a\right)} & =-\frac{U^{\left(p\right)}}{r}f\left(h/r\right)p_{y}+\Omega_{{\hat{x}}^{\prime }}^{\left(a\right)}\sqrt{1-p_{z}^{2}}, \end{array}$$

and

(9) $$\dot{h}=U_{z}^{\left(f,a\right)}=U_{z}^{\left(f,a\right)}\left(p_{z},h/r\right)$$

Note that we used $p_{x}^{2}+p_{y}^{2}+p_{z}^{2}=1$ and $U_{z}^{\left(f,a\right)}$ is the velocity of the particle in the z direction (in problem (f,a)).

In problem (f,a), the particle can move along the surface with constant height and orientation ($\dot{p}=0$ and $\dot{h}=0$) if it is at its fixed points $\left(p_{fixed},h_{fixed}\right)$ [14]. The three fixed points of Equations (6), (7), (8), (9) are given in the Supporting Information Section S5. To refer to the three fixed points, here we use the same nomenclature as reference (log‐rolling and planar alignment), except we refer to the motion in the direction of the external force as force‐taxis (instead of rheotaxis used in the reference) [14]. We call a fixed point stable if the swimmer can reorient toward the fixed point upon introducing a small deviation from the fixed point. [14] So far the dynamical model for problem (f,a) is general and does not depend on the specific details of the propulsion. In order to understand the stability of the fixed points in the problem (f,a), we show that it is possible to consider an alternative experimentally feasible and investigated experiment with the same dynamics.

A similar dynamics to problem (f) applies to the problem of a passive colloid in a *shear* flow (problem (s)), where the translational and rotational velocities, $U^{\left(s\right)}$ and $\Omega^{\left(s\right)}$, of the particle are given by

(10) $$\begin{array}{cc} \Omega^{\left(s\right)} & =-\frac{U^{\left(s\right)}}{h}f^{*}\left(h/r\right)\hat{x}, \end{array}$$

(11) $$\begin{array}{cc} U^{\left(s\right)} & =\dot{\gamma }h\;g^{*}\left(h/r\right)\hat{y}, \end{array}$$

where $\dot{\gamma }$ is the shear rate, and h is the distance between particle's center and the substrate. Since we are ultimately interested in the moving orientation of the active particles, we proceed by analyzing only the similarities in the rotational velocity of the particle between problems (f) and (s). By equating Eqs. (3) and (10), we can find the condition required for the two problems to have the same rotational velocity as

(12) $$U_{\text{equal}}^{\left(s\right)}=U^{\left(f\right)}\left(\frac{h}{r}\right)\left(\frac{f(h/r)}{f^{*}\left(h/r\right)}\right)$$

Hence, by adjusting the particle's velocity in the shear flow to $U_{equal}^{\left(f\right)}$, we can achieve the same vector of rotational velocity as in problem (f). Katuri et al. used the superposition of the problem (s) and a swimmer on a horizontal substrate (problem (a)) to explain the dynamics of active particles' movement in a shear flow. Similar to our discussion, they concluded that, irrespective of the propulsion mechanism, the swimmer has three fixed points, i.e., three modes of swimming in which the swimmer does not change its orientation or height relative to the substrate [14]. As problems (f) and (s) yield the same dynamics of rotational motion, problem (f,a) has the same fixed points as problem (s,a) for the same type of swimmer. Considering problem (f,a), in the following we demonstrate that Pt@SiO_2_ and Cu@SiO_2_ particles are expected to assume cross migration (planar fixed point) and toward‐the‐applied force (force‐taxis fixed point) orientation, respectively. Katuri et al. [14] showed that for problem (s,a) cross‐migration (planar orientation) is the stable mode for Pt@SiO_2_ particles; that is, if the particles' orientation is slightly perturbed, they return to this cross‐migration orientation. Cu@SiO_2_ particles instead align and maintain their orientation in the flow direction, which was confirmed numerically as the stable mode of motion for cap‐forward moving particles [23].

From the equivalency of the problems (s,a) and (f,a), we expect the Pt@SiO_2_ particles to orient and maintain their direction non‐parallel to the direction of the applied force. Therefore, the orientation of the Pt@SiO_2_ particles is dictated by the competition between a gravitational torque aligning particles with the direction defined by the gravity (problem (t)) and hydrodynamically induced rotation (problem (f,a)) opposing the gravitational torque, leading to the low k value for Pt Janus particles compared to the theoretical expectation and Cu particles. Following a similar argument, the Cu@SiO_2_ particles orientation is determined by the superimposed effects of problem (f,a) and problem (t). Since the Cu@SiO_2_ particles in the problem (s,a) orient in the flow direction, the combined effect of problem (f,a) and (t) amplifies the stable orientation in the direction defined by the gravity. This agrees with the higher k values obtained for the Cu Janus particles in our measurements (Figure 3).

### 2.3 Application

Considering the different gravitactic dynamics exhibited by our active particles, we propose two potential applications and show their viability: Particle sorting and a microscopic Galton board for generating a Gaussian random distribution.

**Particle Sorting**: The isolation and purification of specific compounds from heterogeneous samples is important, particularly in biomedical and clinical applications [38, 39]. Microfluidic devices in particular have been promising, reducing the number of samples and reagents but require special designs, fabrication facilities and expensive devices for controlling the flow [39, 40, 41]. Most existing particle and cell separation strategies, whether macro‐ or microfluidic, rely on a well‐defined physical or biochemical contrast such as size, density, deformability, electrical or magnetic properties, or specific binding interactions [42, 43, 44].

**Figure 4:**
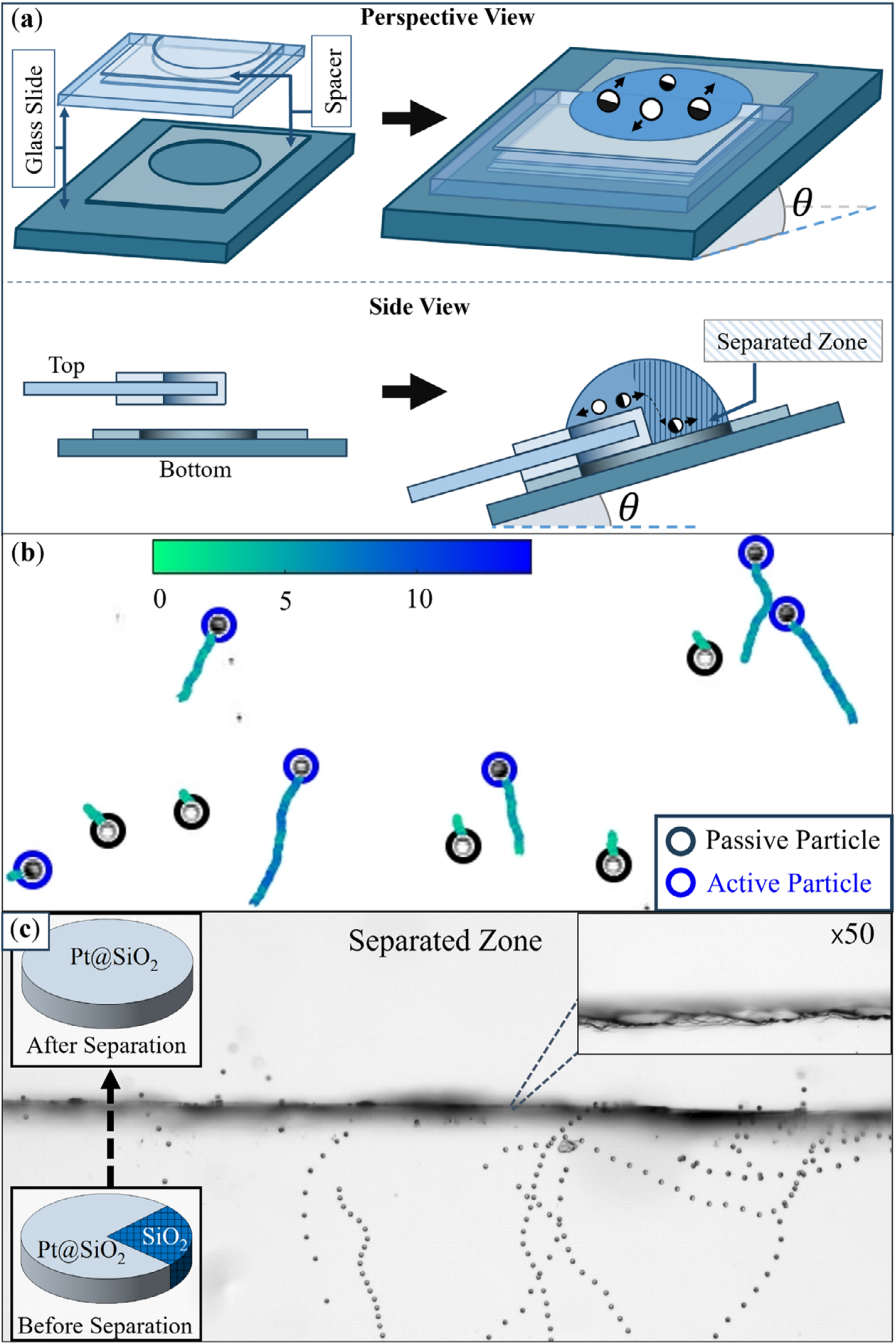
Separating passive and active particles based on their gravitactic behavior. (a) Schematic representation of the setup. A mixture of active and passive particles is initially placed on the upper (blue) glass slide. Upon introducing $H_{2}O_{2}$, active particles migrate upward and, after crossing the edge of the blue slide, fall into the separated zone on the lower (green) glass slide (shown as the hatched region of the water droplet in the side view). (b) Trajectory and speed of passive silica and active Pt@SiO_2_ particles. (c) Trajectory of active particles as a sequence of their images in time as they pass the separating boundary and fall into the other region containing only active particles.

Here we suggest a simple setup allowing the separation of the active particles of different gravitactic behavior (positive and negative) or active and passive colloids. The setup consists of two stacked glass slides immersed in a fluid solution and fixed at a non‐horizontal orientation (Figure 4a). In Figure 4a, the two glass slides are shown schematically, with colors blue and green to distinguish between the top and bottom glass slides, respectively. We place a mixture of passive silica and Pt@SiO_2_ particles of the same size on the top (blue) glass slide, creating a zone that initially contains both species (corresponding to the non‐hatched area of the water droplet in the side view of Figure 4a). At this step, all the particles begin to slowly move downward, toward the boundaries of the top (blue) glass slide. Upon addition of hydrogen peroxide, the Janus particles start moving upward, against the direction defined by gravity (Figure 4b). After passing the edge of the top glass slide, the active particles sediment at the bottom glass slide (corresponding to the hatched area of the water droplet in the side view of Figure 4a). Overlaid snapshots of the particles near the edge are shown in Figure 4c. Some particles may deviate from their upward trajectory for a short distance before crossing the edge of the glass due to irregularities at the edge. Finally, the height difference between the two glasses permanently separates the active particles that pass the edge.

**Figure 5:**
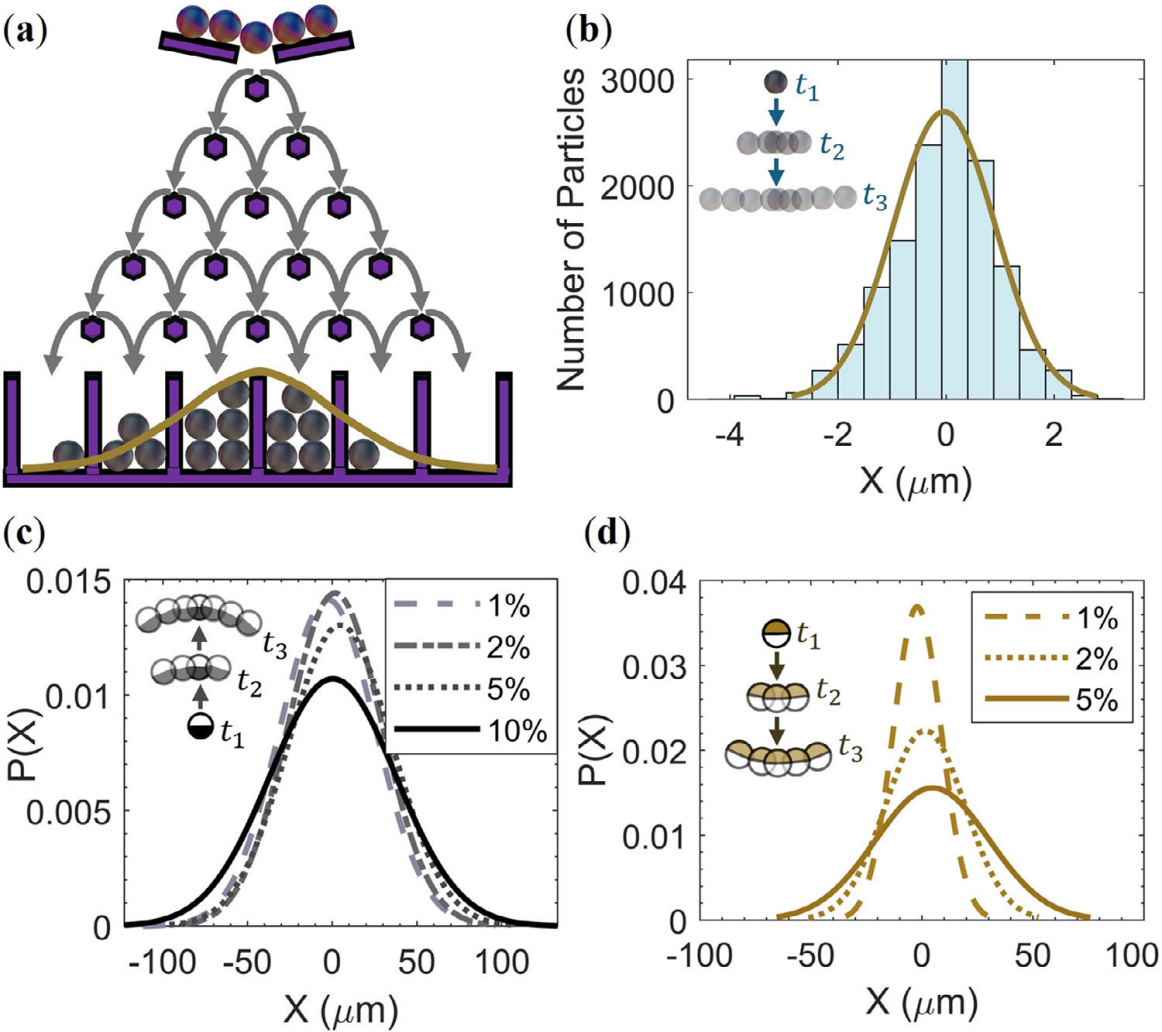
(a) Schematic of the Galton board. (b) Distribution of passive silica particles sliding down a wall, with a fitted Gaussian curve analogous to the Galton board. (c) Fitted Gaussian probability distribution of Pt@SiO_2_ particles at various $H_{2}O_{2}$ concentrations. (d) Fitted Gaussian probability distribution of Cu@SiO_2_ particles at various $H_{2}O_{2}$ concentrations.

**Microscopic Galton Board**: The Galton board is a mechanical device that visually demonstrates the emergence of normal distribution from random processes [45]. It consists of a vertical board with parallel rows of pegs, shown in Figure 5a. Balls are thrown one by one from the top of the board, hitting pegs in each row and randomly moving either to the right or left as they travel to the bottom. The balls are then collected into separate bins, revealing their distribution. This device has been used as a teaching tool and as an illustration scheme for other random processes in many studies [45, 46, 47, 48, 49]. Recent work has highlighted colloidal and active‐matter systems as candidates for unconventional computing, where colloidal suspensions have been used as physical reservoirs for reservoir computing [50, 51, 52, 53]. Maslen et al. further demonstrated that the Brownian orientation of microscopic rods can serve as a physical implementation of Monte Carlo sampling to compute π [53].

Analogous to the Galton board, sedimenting microscopic particles on a tilted substrate can generate a Gaussian distribution. At each small time step Δt, each colloid moves randomly to the left or right, with a displacement proportional to $\sqrt{t}$. Figure 5b shows the horizontal distribution of passive SiO_2_ particles sliding down the substrate over 7.5 s. A similar experiment can be conducted with gravitactic Janus particles. In this case, in addition to the translational Brownian motion, an additional contribution, $V_{0}\sin\alpha \Delta t$, arises from the rotational diffusion of the colloids, introducing a deviation angle α between the particle and the y axis. Figure 5c,d shows the fitted Gaussian distributions for Pt and Cu Janus particles in different concentrations of the peroxide. Generally, the distribution broadens for both types of active colloids as the peroxide concentration increases. This is expected as the higher activity of the particles results in larger random walks at each time step. Moreover, Cu Janus particles exhibit a narrower distribution compared to Pt Janus particles, reflecting their better alignment in the direction of gravity.

As discussed here, both passive and active colloids produce characteristic stochastic distributions when moving on a tilted substrate. By binning the particles laterally, we derived a distribution from the counts in each bin, directly analogous to the output of a Galton board. This approach reveals how the activity and gravitactic behavior of the particles influence the sampling statistics, and provides control over the sedimentation direction, which is crucial for understanding how active‐matter systems could be tuned for future physical computing architectures.

## 3 Conclusion

In this study, we investigated the gravity‐induced dynamics of Pt@SiO_2_ and Cu@SiO_2_ Janus particles on an inclined substrate. We confirmed the negative gravitaxis of Pt@SiO_2_ particles, consistent with previous reports, and demonstrated positive gravitaxis in Cu@SiO_2_ particles, which arises from their distinct cap‐forward propulsion mechanism. Notably, Cu@SiO_2_ particles exhibited stronger alignment with the gravity‐defined direction compared to Pt@SiO_2_, despite the lower density of copper (compared to Pt).

Our results further reveal that hydrodynamic interactions with the wall contribute significantly to particle orientation. Owing to their proximity to the substrate, the gravitational force component parallel to the surface generates a hydrodynamic torque that aligns Cu@SiO_2_ particles along the sedimentation direction, whereas Pt@SiO_2_ particles orient perpendicular to it.

Utilizing the distinct gravitactic behaviors of passive and active particles, we demonstrated a simple particle sorting method based on their gravitactic dynamics. Additionally, we observed that the lateral sedimentation profiles of both passive and active particles follow a Gaussian distribution, with the variance indicating the degree to which active motion is quenched along the direction of gravity. The passive and active Brownian downward or upward motion of particles on an inclined substrate resembles a microscopic‐scale Galton board and presents opportunities for educational demonstrations and computational applications.

## 4 Experimental Section

### 4.1 Preparation of Pt@SiO_2_ and Cu@SiO_2_ Janus colloids

In order to obtain Janus colloids, drops of a suspension of spherical SiO_2_ (5 μm diameter, Sigma–Aldrich) particles in absolute ethanol (≥99.8%, Fisher Chemical, Germany) were evenly spread on a plasma‐cleaned glass slide. Plasma‐cleaned glass slides were prepared by treating the slides with an $O_{2}$ plasma at 0.3–0.4 mbar for 3.5 min using a Diener Electronic Zepto plasma cleaner. This was followed by slow solvent evaporation, to form a monolayer. Next, a 30 nm Pt layer was deposited on the monolayer using an e‐beam system (or 30 nm Cu was deposited in the case of Cu@SiO_2_). Sputtering of Pt (or Cu) was done using a Quorum Q150T ES sputter coater with a Pt (or Cu) target at a pressure of 10^−2^ mbar (Ar). Finally, the Janus colloids were released in de‐ionized water using short ultrasonic pulses. All the experiments are performed at room temperature.

### 4.2 Motion Studies of Pt@SiO_2_ and Cu@SiO_2_ Particles at the horizontal substrate

Motion studies were performed on a cleaned‐glass slide attached to the end of a glass capillary (both cleaned with acetone, and ethanol, followed by plasma treatment). Subsequently, 1μL aqueous suspension of either Pt@SiO_2_ or Cu@SiO_2_ particles was placed on the cleaned glass substrate. After allowing particles to sediment, a 500μL $H_{2}O_{2}$ (Fisher Chemical, Thermo Fisher Scientific) solution was added. Conductivity and pH values of H_2_O_2_ solutions at different concentrations are represented in Supporting Information Section S9. The motion of the particles was recorded at 40 frames per second with an image size of 960×608 pixels.

### 4.3 Motion Studies of Pt@SiO_2_ and Cu@SiO_2_ Particles at the Inclined Substrate

In the case of the gravitaxis study, the microscope (Carl Zeiss inverted microscope, Axio Observer 5) was placed on a homemade triangular ramp with a defined angle of 15 degrees. Motion studies were performed on a cleaned‐glass slide attached to the end of a capillary (both cleaned with acetone, and ethanol, followed by plasma treatment). Next, 1 μL aqueous suspension of either Pt@SiO_2_ or Cu@SiO_2_ particles was placed on the cleaned glass substrate. After allowing particles to sediment, a 500μL $H_{2}O_{2}$ solution was added. Subsequently, the motion of the particles was recorded at 40 frames per second.

### 4.4 Video Recording and Speed Analysis

All the videos were recorded using a Zeiss camera (Axiocam 702 Mono) attached to a Carl Zeiss inverted microscope equipped with a 20x N‐Acroplan objective (depth of focus: 5.43 μm). No additional digital zoom was applied. Before video acquisition, the illumination of the sample was adjusted manually to achieve optimal particle‐background contrast. Each velocity value and each fitted k parameter were computed from at least 9000 data points, corresponding to at least 30 particles tracked over 300 frames from a minimum of four videos. The videos were analyzed using homemade MATLAB (R2019b) code to yield trajectories with x–y coordinates, which were further processed for calculating instantaneous speeds every frame using the distance between two Cartesian coordinates formula. Finally, the tracks with the instantaneous speeds were plotted.

### 4.5 Angle Analysis

The in‐plane angle α was calculated in MATLAB following the method described by Wang et al. [54]. Briefly, the centers of the entire colloid and its metal‐coated region were identified using custom MATLAB code after thresholding of the video frames using the intensity value of 100 (with pixel intensities ranging from 0 to 255). The in‐plane angle was then computed using the formula $\alpha =\arctan(y_{0}-y_{1},x_{0}-x_{1})$, where $\left(x_{0},y_{0}\right)$ and $\left(x_{1},y_{1}\right)$ denote the coordinates of the center of the whole colloid and the metal‐coated region, respectively. To obtain the concentration parameter k, Von Mises distributions (Equation 2) were fitted using MATLAB's nonlinear least‐squares routine (fit). MATLAB's built‐in confint function was used to obtain the 95% confidence intervals for the fitted parameters. The k values and their 95% confidence intervals are tabulated in the Supporting Information Section 7.

### 4.6 SEM

For SEM imaging, dilute dispersions of Pt@SiO_2_ or Cu@SiO_2_ microparticles in ethanol were drop‐casted onto silicon wafer pieces and attached to specimen support with conductive carbon tape. Samples were dried overnight and imaged on a Zeiss Ultra Plus microscope with an acceleration voltage of 3 kV.

## Conflicts of Interest

The authors declare no conflict of interest.

## Supporting information




**Supporting File**: smll72072‐sup‐0001‐SuppMat.pdf

**Supporting Movie**: smll72072‐sup‐0002‐MovieS1.avi

**Supporting Movie**: smll72072‐sup‐0003‐MovieS2.avi

**Supporting Movie**: smll72072‐sup‐0004‐MovieS3.avi

## Data Availability

The data that support the findings of this study are available in the supplementary material of this article.
