# Supplementary material for: Navigating Gravity: Competing Effects Result in Opposing Taxis for Different Janus Swimmers
Source: Small. 2025 Dec 28;22(10):e08984. doi: 10.1002/smll.202508984 (PMC12910435; doi:10.1002/smll.202508984)
Supplement: Supplementary file 1 — Supporting File: smll72072‐sup‐0001‐SuppMat.pdf [file SMLL-22-e08984-s001.pdf]

Navigating Gravity: Competing Effects result in Opposing Taxes for Different Janus Swimmers

Amir Sheikh Shoaei[1], Jens-Uwe Sommer[2], Juliane Simmchen[3*]

[1]Technische Universität Dresden, Chair of Physical Chemistry, 01062 Dresden, Germany

[2]Technische Universität Dresden, Department of Physics, 01062 Dresden, Germany

[3]University of Strathclyde, Pure and Applied Chemistry, G1 1XL Glasgow, United Kingdom

December 22, 2025

1 List of Videos

Video 1: Pt@SiO_2 Janus particles on horizontal and tilted substrates.
Video 2: Cu@SiO_2 Janus particles on horizontal and tilted substrates.
Video 3: Separation of passive (SiO_2) and active (Pt@SiO_2) particle.

2 Velocity and orientation on a horizontal substrate

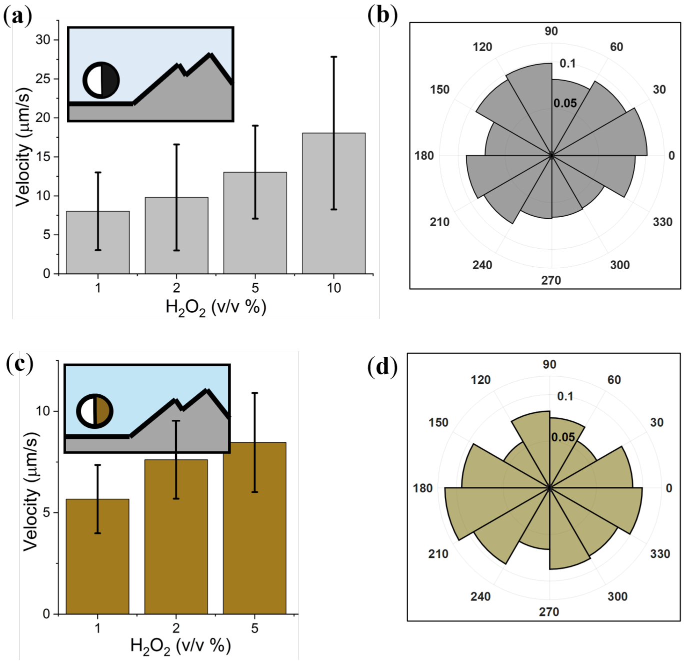

Figure 1: Velocity and orientation distributions of (a) and (b) Pt@SiO_2, and (c) and (d) Cu@SiO_2 Janus particles.

3 Velocity of passive particles on tilted substrate

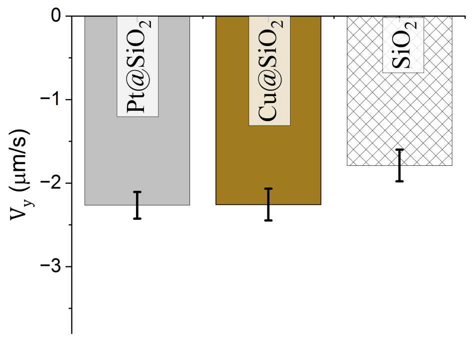

Figure 2: Velocity of passive SiO_2, Pt@SiO_2, and Cu@SiO_2 (in Deionized water) on Tilted Substrate.

4 Egg-shell model and the direction of movement of the particles

To measure gravitational torque on the Janus particles, we need to determine the center of mass (COM) of the particle as well as the mass of the cap. Here we follow the model presented by Campbell et al[1]. In this model, the catalytic cap is considered to have an ellipsoidal shape with the maximum thickness at the center of the cap and going to zero thickness towards the sphere equator (figure 3). The center of mass is located at the distance $l = \frac{3}{4}\left(R + \frac{1}{2}l_{\text{cap}}\right) \approx \frac{3}{4}R$, where l_{cap} is the thickness of the catalytic cap. Furthermore, the mass of the cap can be obtained as $m_a = \frac{2\pi}{3}l_{cap}\rho_a$, where ρ_a is the density of the active cap.

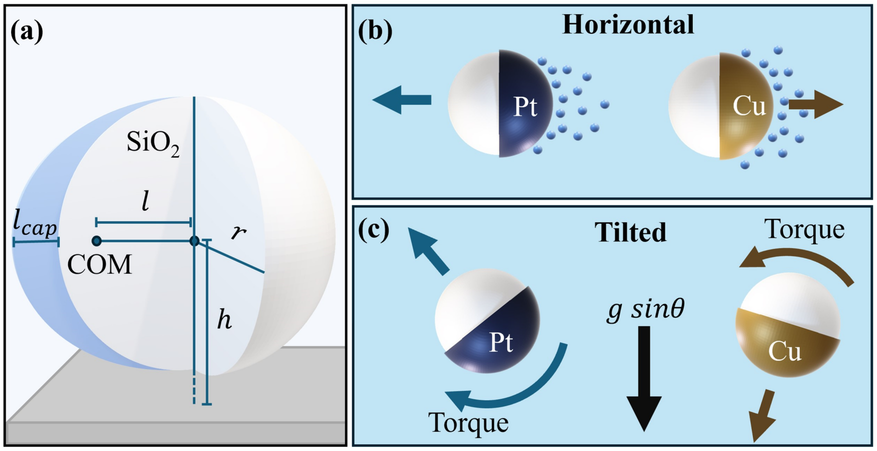

Figure 3: (a) schematic representation of the egg-shell model for a Janus particle with a catalytic layer covering half of the silica core, where the thickness of the catalytic cap is maximum in the center and goes to zero on the boundaries. The direction of the movement of the Pt@SiO_2 and Cu@SiO_2 on (b) horizontal and (c) a tilted substrate, where the black arrow shows the direction of the gravity and its contribution inside the tilted plane.

5 Functions $f(h/r)$ and $g(h/r)$

The equations for the functions $f(h/r)$ and $g(h/r)$ are derived from[2] calculations as

$$f\left(\frac{\delta}{r}\right) = \frac{\ln(\delta/a)}{4\ln(\delta/a) - 3.817}, \tag{1}$$

$$g\left(\frac{\delta}{r}\right) = \frac{2\ln(\delta/a) - 1.9086}{[\ln(\delta/a)]^2 - 4.325\ln(\delta/a) + 1.591}. \tag{2}$$

where $\delta = h - 1$ is the closest distance between a point on the particle and the substrate.

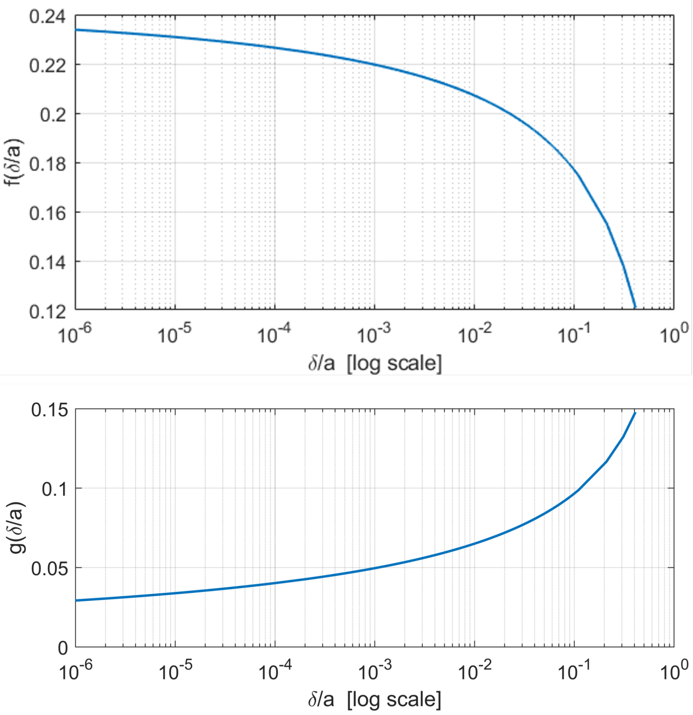

Figure 4: Functions $f(\delta/r)$ and $g(\delta/r)$, representing the influence of the wall on the hydrodynamic coupling between translational and rotational velocity and the ratio of the force exerted on the particle in the proximity of the wall as compared to the bulk, respectively. Note that here we used $\delta = h - 1$ for convenience.

6 Configuration of the swimmer corresponding to the fixed points

Using equations 6 to 9 in the main text, we can find the fixed points $((p_{fixed}, h_{fixed}))$ that satisfy the condition $\dot{\boldsymbol{p}} = 0$ and $\dot{h} = 0$. To achieve $\dot{p}_x^{(f,a)} = -\frac{\Omega^{(a)}_{\hat{x}'} p_x p_z}{\sqrt{1-p_z^2}} = 0$, we have three possibilities, namely, $\Omega^{(a)}_{\hat{x}'} = 0$, $p_z = 0$ or $p_x = 0$. If $\Omega^{(a)}_{\hat{x}'} = 0$ we further require $p_z = p_x = 0$. This configuration corresponds to log-rolling in figure 5 a. In the cases $p_z = 0$ and $p_x = 0$, we further need $\Omega^{(a)}_{\hat{x}'}(p_z, h/r) = \frac{U^{(p)}}{r} f(h/r)$. The configurations planar alignment and force-taxis shown in figure 5 b and c correspond to $p_z = 0$ and $p_x = 0$, respectively. Note that log-rolling is a special case of planar alignment.

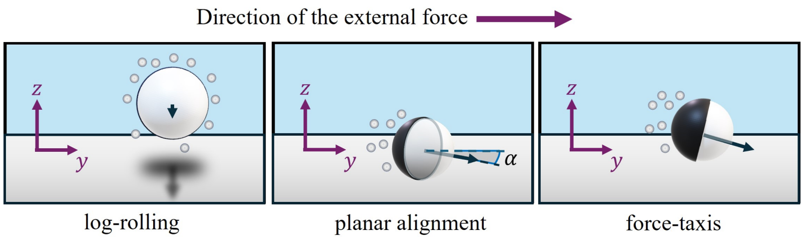

Figure 5: Three configurations in which a swimmer can have constant orientation and height as it moves. Here we use a similar argument and nomenclature as reference [3] (a) The swimmer's direction remains parallel to the x direction (log-rolling). (b) In the planar alignment configuration, the swimmer's direction is in the x-y plane ($p_z = 0$). Due to symmetry, this configuration happens in pairs ($p_x = \pm p_{x,planar}$. (c) The direction of the swimmer is in the z-y plane, the plane defined by the force and a vector normal to the substrate.

7 Velocity Statistics and k Values on the Tilted Substrate

Pt@SiO$_2$				
H_2O_2 (v/v %)	Mean velocity (μm/s)	SD (μm/s)	k	95% CI for
1	1.62	3.93	1.78	(1.74,1.82)
2	2.83	4.06	1.84	(1.81,1.87)
5	5.30	7.63	2.32	(2.23,2.40)
10	5.29	6.83	2.25	(2.17,2.34)

Figure 6: Mean velocities, standard deviations, and k values with their corresponding 95% confidence intervals for Pt@SiO$_2$ particles on a tilted substrate.

Cu@SiO$_2$				
H_2O_2 (v/v %)	Mean velocity (μm/s)	SD (μm/s)	k	95% CI for
1	-7.83	2.74	12.85	(12.31,13.40)
2	-10.24	2.41	10.57	(10.06,11.08)
5	-10.71	5.86	3.76	(3.67,3.85)

Figure 7: Mean velocities, standard deviations, and k values with their corresponding 95% confidence intervals for Cu@SiO$_2$ particles on a tilted substrate.

8 Velocity and k values on the tilted substrate with different angles

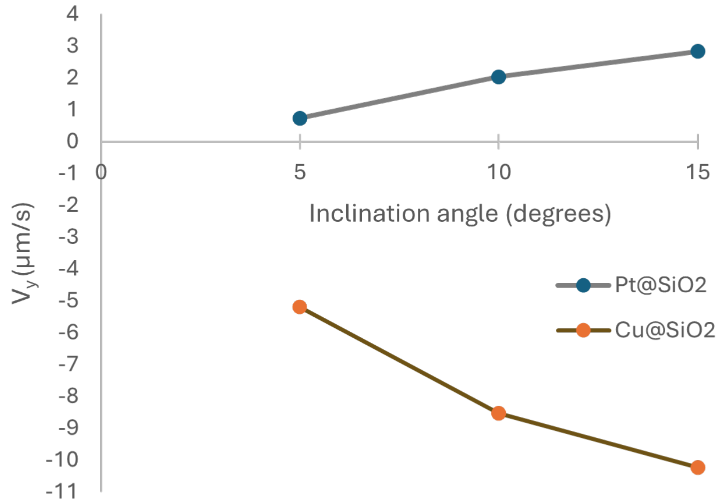

Figure 8: Mean velocity of Pt@SiO_2 and Cu@SiO_2 particles moving in a 2% H_2O_2 solution on a tilted substrate at different inclination angles.

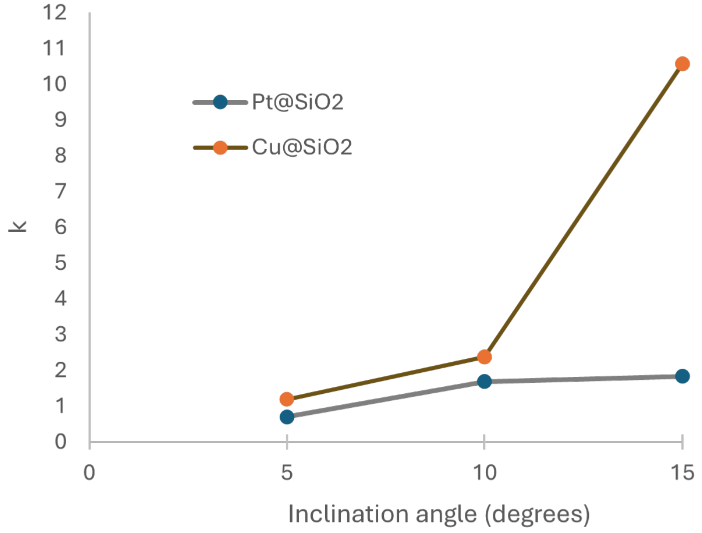

Figure 9: Concentration parameter k of Pt@SiO_2 and Cu@SiO_2 particles moving in a 2% H_2O_2 solution on a tilted substrate at different inclination angles.

9 Conductivity and pH values

Table 1: Conductivity and pH values of H_2O_2 solutions at different concentrations.

H_2O_2 Concentration	Conductivity (µS/cm)	pH
0%	0.6	6.7
1%	1.2	6
2%	1.55	5.8
5%	1.58	5.4

10 Estimate of the Lever Arm for a Metal-Capped Janus Particle

In our sputtering procedure, the deposition system is calibrated to deposit a nominal thickness of approximately 30 nm of metal (Cu or Pt) on a flat surface. For spherical particles, the total deposited mass is primarily governed by the projected shadow area, πr^2, during line-of-sight sputtering. Consequently, the *total deposited mass* is expected to be similar for both metals, and differences between geometrical models affect mainly the *lever arm* l, rather than the total mass.

To quantify this sensitivity, we examine how l varies with the assumed thickness profile. Experimental studies consistently report that sputtered metal caps become thinner toward the equator of the particle[4,1,5,6]. Since the deposited layer ($\sim$ 30 nm) is much smaller than the particle radius ($\sim$ 2500 nm), the center of mass of the deposited material must lie within the particle's radius, such that

$$l < r.$$

To estimate a lower bound for l, we consider a hypothetical geometry in which the deposited cap has a uniform thickness over the entire hemisphere. The center of mass of a hollow hemisphere of radius r lies at $r/2$ from its pole. Therefore, for realistic thickness profiles, one expects the lever arm to satisfy

$$\frac{r}{2} < l < r.$$

The eggshell model used in the main manuscript yields $l = 0.75\,r$, placing it at the midpoint of this interval. Hence, the uncertainty associated with the choice of geometrical model corresponds to a relative variation of approximately $\pm 33\%$ in the lever arm l.

References

[1] A. I. Campbell, S. J. Ebbens, *Langmuir* **2013**, *29*, 46 14066.

[2] A. J. Goldman, R. G. Cox, H. Brenner, *Chemical engineering science* **1967**, *22*, 4 637.

[3] J. Katuri, W. E. Uspal, J. Simmchen, A. Miguel-López, S. Sánchez, *Science advances* **2018**, *4*, 1 eaao1755.

[4] P. Sharan, Z. Xiao, V. Mancuso, W. E. Uspal, J. Simmchen, *ACS nano* **2022**, *16*, 3 4599.

[5] S. Das, Z. Jalilvand, M. N. Popescu, W. E. Uspal, S. Dietrich, I. Kretzschmar, *Langmuir* **2020**, *36*, 25 7133.

[6] A. Rashidi, M. W. Issa, I. T. Martin, A. Avishai, S. Razavi, C. L. Wirth, *ACS Applied Materials & Interfaces* **2018**, *10*, 37 30925.
